# *Sphingomonas taxi*, Isolated from *Cucurbita pepo*, Proves to Be a DDE-Degrading and Plant Growth-Promoting Strain

**DOI:** 10.1128/genomeA.00489-15

**Published:** 2015-05-14

**Authors:** Nele Eevers, Jonathan D. Van Hamme, Eric M. Bottos, Nele Weyens, Jaco Vangronsveld

**Affiliations:** a Centre for Environmental Sciences, Hasselt University, Diepenbeek, Belgium; b Department of Biological Sciences, Thompson Rivers University, Kamloops, British Columbia, Canada

## Abstract

The draft genome of *Sphingomonas taxi*, a strain of the *Sphingomonadaceae* isolated from *Cucurbita pepo* root tissue, is presented. This Gram-negative bacterium shows 2,2-bis(*p*-chlorophenyl)-1,1-dichloroethylene (DDE)-degrading potential and plant growth-promoting capacities. An analysis of its 3.9-Mb draft genome will enhance the understanding of DDE-degradation pathways and phytoremediation applications for DDE-contaminated soils.

## GENOME ANNOUNCEMENT

DDT (2,2-bis[*p*-chlorophenyl]-1,1,1-trichloroethane) (1) is an agricultural and gardening pesticide that has been used since 1943 (2). When exposed to soil conditions, DDT degrades to 2,2-bis(*p*-chlorophenyl)-1,1-dichloroethylene (DDE). These products are categorized as persistent organic pollutants (POPs) (3) and threaten human and wildlife health because of their bioaccumulative and hormone-disrupting properties (4). DDE phytoremediation using *Cucurbita pepo*, a DDE accumulator (5), in combination with suitable endophytes, may resolve problems associated with DDE-contaminated soils.

The *Sphingomonas taxi* strain described here was isolated from *Cucurbita pepo* root tissue that was exposed to 100 mg·liter^−1^ of DDE during cultivation. Partial 16S rRNA gene sequencing and phenotypic profiling identified the strain as *Sphingomonas taxi*, with the closest related 16S rRNA sequence (87%) being from strain ATCC 55669 (GenBank accession no. CP009571.1).

To better characterize the isolate, DNA was extracted, and the whole-genome shotgun sequence was prepared on an IonTorrent PGM, as described by Eevers et al. (6).

In all, 1.29 million reads with a mean length of 270 bases generated 349 Mb of data in Torrent Suite version 4.2.1. Assembly using SPAdes version 3.1.0 (7, 8) (uniform coverage mode; *k*-mers 21, 33, 55, 77, 99) yielded 109 contigs >1,000 bp, giving a consensus length of 3,941,497 bp at a 51× coverage (largest contig, 231,989 bp; *N*_50_, 75,931 bp). The contigs were ordered with Mauve (9), using the *Sphingomonas* taxi ATCC 55669 genome (accession no. CP00951.1) as a reference. The PGAP (NCBI) pipeline was used for annotation (10). The *S. taxi* genome consists of a single circular chromosome (67.15% GC content), which includes 534 pseudogenes, 3 rRNAs (5S, 16S, 23S), 48 tRNAs, and 1 noncoding RNA (ncRNA), and 3,634 coding genes that were arranged into 378 pathways using Pathway Tools (11, 12).

In experiments testing DDE-degrading capacities, *Sphingomonas taxi* showed increased growth when exposed to 50 mg·liter^−1^ DDE in comparison to control conditions. This result is in agreement with the presence of halogenases, dioxygenases, and hydrolases (13–16). The presence of pathways related to the degradation of phenylacetate, octane, acrylonitrile, toluene, phenylmercury acetate, and naphthalene, as well as for the detoxification of arsenate and superoxide radicals, make this *Sphingomonas taxi* strain a suitable candidate for the phytoremediation of soils polluted with mixed contaminants. Genes coding for plant growth-promoting capacities are also present, confirming results from phenotypic tests: 1-aminocyclopropane-1-carboxylate deaminase activity, siderophore production, auxin biosynthesis, and phosphorous solubilization. Interestingly, pathways for nitrogen and carbon dioxide fixation are present, although we have not confirmed these traits. The strain also shows a capacity for hemicellulose and cellulose degradation, a useful trait for facilitating entrance into plant roots during inoculation. This combination of characteristics makes *Sphingomonas taxi* a promising strain for the phytoremediation of soils contaminated with DDE and other contaminants.

### Nucleotide sequence accession numbers.

This whole-genome shotgun project has been deposited at DDBJ/EMBL/GenBank under the accession number JXTP00000000. The version described in this paper is version JTXP01000000.
